# Nanostructured Coatings Based on Graphene Oxide for the Management of Periprosthetic Infections

**DOI:** 10.3390/ijms25042389

**Published:** 2024-02-17

**Authors:** Sorin Constantinescu, Adelina-Gabriela Niculescu, Ariana Hudiță, Valentina Grumezescu, Dragoș Rădulescu, Alexandra Cătălina Bîrcă, Gabriela Dorcioman, Oana Gherasim, Alina Maria Holban, Bianca Gălățeanu, Bogdan Ștefan Vasile, Alexandru Mihai Grumezescu, Alexandra Bolocan, Radu Rădulescu

**Affiliations:** 1 Faculty of Medicine, Carol Davila University of Medicine and Pharmacy, 8 Eroii Sanitari Street, 050474 Bucharest, Romania; dr.sorin.c@gmail.com (S.C.); dragos.radulescu@umfcd.ro (D.R.); alexandra.bolocan@umfcd.ro (A.B.); radu_radulescu@umfcd.ro (R.R.); 2 Research Institute of the University of Bucharest—ICUB, University of Bucharest, 90-92 Panduri, 050663 Bucharest, Romania; adelina.niculescu@upb.ro (A.-G.N.); ariana.hudita@bio.unibuc.ro (A.H.); alina.m.holban@bio.unibuc.ro (A.M.H.); 3 Department of Science and Engineering of Oxide Materials and Nanomaterials, University Politehnica of Bucharest, 1-7 Gh. Polizu Street, 060042 Bucharest, Romania; alexandra.birca@upb.ro (A.C.B.); bogdan.vasile@upb.ro (B.Ș.V.); 4 Department of Biochemistry and Molecular Biology, University of Bucharest, 91-95 Splaiul Independentei Street, 050095 Bucharest, Romania; bianca.galateanu@bio.unibuc.ro; 5 Lasers Department, National Institute for Lasers, Plasma and Radiation Physics, 409 Atomistilor Street, 077125 Magurele, Romania; valentina.grumezescu@inflpr.ro (V.G.); gabriela.dorcioman@inflpr.ro (G.D.); oana.gherasim@inflpr.ro (O.G.); 6 Microbiology and Immunology Department, Faculty of Biology, University of Bucharest, 1-3 Portocalelor Lane, 77206 Bucharest, Romania

**Keywords:** polylactide (PLA), graphene oxide, MAPLE, nanostructured coatings, hemocompatible coatings, anti-biofilm efficiency

## Abstract

To modulate the bioactivity and boost the therapeutic outcome of implantable metallic devices, biodegradable coatings based on polylactide (PLA) and graphene oxide nanosheets (nGOs) loaded with Zinforo™ (Zin) have been proposed in this study as innovative alternatives for the local management of biofilm-associated periprosthetic infections. Using a modified Hummers protocol, high-purity and ultra-thin nGOs have been obtained, as evidenced by X-ray diffraction (XRD) and transmission electron microscopy (TEM) investigations. The matrix-assisted pulsed laser evaporation (MAPLE) technique has been successfully employed to obtain the PLA-nGO-Zin coatings. The stoichiometric and uniform transfer was revealed by infrared microscopy (IRM) and scanning electron microscopy (SEM) studies. In vitro evaluation, performed on fresh blood samples, has shown the excellent hemocompatibility of PLA-nGO-Zin-coated samples (with a hemolytic index of 1.15%), together with their anti-inflammatory ability. Moreover, the PLA-nGO-Zin coatings significantly inhibited the development of mature bacterial biofilms, inducing important anti-biofilm efficiency in the as-coated samples. The herein-reported results evidence the promising potential of PLA-nGO-Zin coatings to be used for the biocompatible and antimicrobial surface modification of metallic implants.

## 1. Introduction

Bacterial biofilms represent an increasing concern for patients, health professionals, and healthcare providers worldwide, especially due to the emergence and alarming spread of antimicrobial-resistant pathogens and taking into consideration the high demand for implantable medical devices (IMDs) [1,2,3]. Compared to planktonic cells, biofilm-embedded microorganisms form a complex adaptive system and possess a much higher intrinsic antimicrobial resistance, displaying different growth and spreading rates, particular structural and functional characteristics, versatile protective mechanisms, altered pathogenicity factors, and genetic mutations, therefore rendering their eradication far more challenging [4,5,6]. In more detail, sessile bacteria (biofilm-forming cells) tolerate antibiotics, ultraviolet light, chemical biocides, host immune response, and harsh environmental conditions (e.g., extreme temperatures and pH, high salinity and pressure, poor nutrients) well [1,7].

As the surfaces of IMDs are particularly prevalent sources of bacterial contamination (and subsequent colonization), biofilms reportedly contribute to ~65% of nosocomial infections, including major hospital-acquired infections [8]. Bacterial cells can attach and colonize on the surface of indwelling catheters, mechanical heart valves, pacemakers, endovascular stents, prosthetic joints and implants, voice prosthesis, artificial lenses, and internal or external fixation devices, generating inception infection points [1,9,10,11,12,13,14]. Besides constraining the immune system of the host organism by causing a moderate-to-severe systemic response [15,16], biofilm-associated infections can also affect the structure and functionality of the implanted devices [17], further imposing their removal [2,14].

According to the recent literature, orthopedic interventions have significant rates of post-operative surgical site infection, namely 13% to 88% for tibial plateau fractures, 3% to 45% for proximal tibia fractures, 3% to 17% for distal femur fractures, and 2% to 10% for patellar fractures [18]. Furthermore, 1% to 5% of orthopedic implants have been reported to produce infections, being linked to considerable morbidity, disability, and healthcare costs [19]. Additionally, the use of fixation devices (such as plates, wires, screws, nails, and pins) can increase the risk of infection through biofilm formation and may complicate surgical debridement, resulting in more technically demanding procedures and longer operative times [20]. Even though metals are viewed as gold-standard materials for restorative or reconstructive orthopedic IMDs, they are still associated with periprosthetic infections [21,22], mechanical failure [23,24], local corrosion, and ion-mediated chronic toxicity [25,26], with a greater probability for revision surgery and hardware removal [27,28].

Reconstruction surgery employing tendon autografts is a preferred therapeutic strategy for patients with knee ligamental injuries. Given the essential role of ligaments in knee stability and biomechanics and with the aim of avoiding multiple interventions, special attention must be oriented towards graft positioning and fixation during surgery [29,30]. Metallic (titanium) and composite (biphasic calcium phosphate and polyester copolymer) screws or pins are available as fixation devices for knee ligament reconstruction, providing maximal bone fixation [31,32]. Still, recent data report alarming long-term effects associated with these devices, such as similar joint effusion, inflammation, and infection, but also a higher risk of intra-articular migration and rupture for composite implants [33]. However, artificial polymer-based ligaments have been validated as a therapeutic alternative for extensive ligament injuries, but such devices are fabricated from biocompatible and non-bioactive materials that are susceptible to microbial contamination [34].

The current practice against biofilm-related infection is the systemic administration of multiple and high-dose antibiotics, while prevention is generally ensured by the sterilization of medical surfaces [17]. For instance, advanced-generation cephalosporins (beta-lactam antibiotics) have impressive outcomes in the treatment of major-to-severe infectious diseases, including resistant and nosocomial infections, by inhibiting the penicillin-binding protein-mediated crosslinking of the peptidoglycan layer within the bacterial cell wall [35,36]. Nonetheless, systemic antibiotic therapy may lead to side effects, low patient compliance, development of drug-resistant pathogens, and reduced treatment efficiency [37,38,39]. On the other hand, during the conventional sterilization of surgical instruments and IMDs, the products are decontaminated, washed, reassembled, labeled, sterilized, and redistributed. However, only a small portion of processed implants are used during surgery, leading to multiple reprocessing of the remaining IMDs before being placed in a patient. These practices open the door to preoperative contamination, increasing the infection risk [40].

Thus, given that current anti-infective strategies are often inefficient and partially selective or even threaten the patient’s safety, there is an urgent need for developing improved solutions. One notable and promising approach against persistent bacterial infections is the optimization of clinical IMDs by their surface modification with microbial-repellent or microbial-resistant coatings. Specifically, improving the surfaces of implants and fixation devices with bioactive coatings (that may exert multiple biofunctionalities) has the potential to prevent microbial attachment, intercept biofilm development, and reduce the transmission of pathogens in the clinical environment [8,17,41,42].

Interesting possibilities with high-quality therapeutic outcomes can be envisaged by incorporating conventional (synthetic drugs and natural phytochemicals) and unconventional (nanosized and nanostructured materials) antimicrobials within biodegradable polymer layers that provide sustained local release and facilitate circumstantial controlled and triggered delivery. The use of biopolymers has attracted increasing interest in modern pharmacotherapy, being either explored as efficient delivery and releasing vehicles for antibiotics or natural antimicrobials, or for their intrinsic biological effects. Due to their unique size-governed physicochemical properties, antimicrobial nanomaterials can overcome bacterial resistance as they can permeate and destroy bacterial cell membranes, display microbiostatic or microbicidal effects, and hamper biofilm formation. Thus, their incorporation into coatings for tuning the surfaces of IMDs serves as a performant strategy to prevent or limit microbial adhesion and mitigate or kill the biofilm-embedded microorganisms [3,43,44,45,46,47,48,49,50].

Graphene is a versatile two-dimensional representative of carbon-based nanomaterials, and possesses unique electronic, mechanical, optical, and thermal properties that set up great expectations for technical applications, including biotechnology and biomedicine. Its intrinsic biocompatibility and low toxicity, tunable mechanical support and elasticity for cellular adhesion and migration, excellent stability, and appropriate conductivity for modulated cellular behavior especially recommend this material for use in biosensing and bioimaging platforms, accurate drug delivery vehicles, and scaffold production for tissue engineering and regenerative medicine [51,52,53]. In addition, graphene oxide (GO, representing the chemically modified form of graphene) is recognized for its extensive and versatile surface area and outstanding thermal, mechanical, and electrical properties, while also possessing beneficial biological roles (electroactivity-mediated immunomodulation, molecular regulation, and cellular events—including differentiation and guided cytophysiology) and intrinsic antimicrobial activity [52,54].

Owing to its attractive features (versatile composition, facile and cost-effective processability, adaptable solubility and degradability, and tunable physicochemical and thermomechanical behavior), polylactide (PLA, a thermoplastic naturally derived biopolymer) has been extensively explored for use in innovative therapeutic strategies. In addition, the excellent biocompatibility, non-toxicity, tunable biodegradability, and reduced immunogenicity of PLA-based formulations pave the way for fabricating bioactive platforms for personalized and modern biomedicine [55,56].

Even though PLA is a widely used biopolymer in biotechnology and biomedicine, it still exhibits some drawbacks, including poor mechanical behavior, lack of intrinsic bioactivity, and the absence of antibacterial behavior. Incorporating antimicrobial nanofillers has been proposed to overcome these limitations, as different kinds of additives can synergistically improve the properties of PLA-based composites [57,58]. Among diverse possibilities, GO nanofillers are especially useful additives for producing multifunctional nanocomposites with adequate mechanical strength, improved flexibility, adaptable barrier role, antibacterial activity, and enhanced biocompatibility [58,59].

In this context, this study proposes the fabrication of biocompatible coatings based on polylactide (PLA) and graphene oxide nanosheets (nGOs) for the loading and release of Zinforo™ (Zin). Using the matrix-assisted pulsed laser evaporation (MAPLE) technique, this broad-spectrum cephalosporin antibiotic (a ceftaroline fosamil prodrug) has been successfully transferred within bioactive coating formulations for the surface modification of IMDs. The herein-developed PLA-nGO-Zin nanostructured coatings represent innovative bioactive alternatives for the local management (prevention and limitation) of biofilm-associated periprosthetic infections.

## 2. Results and Discussions

### 2.1. Physicochemical Characterization of GO Nanomaterial

The Hummers method is a multi-step, facile, and high-yield protocol to obtain GO, conventionally by using H_2_SO_4_, sodium nitrate (NaNO_3_), and KMnO_4_ to oxidize graphite. To overcome the hazard of toxic gases related to NaNO_3_ decomposition, different chemicals have been successfully proposed to remove NaNO_3_ during the synthesis [60,61]. To improve the oxidation rate of graphite and achieve GO with better oxidation degree [62], a modified Hummers methodology—consisting in using a mixture of K_2_S_2_O_8_ and P_2_O_5_ instead of the conventional NaNO_3_—was applied in our experiments.

**Figure 1:**
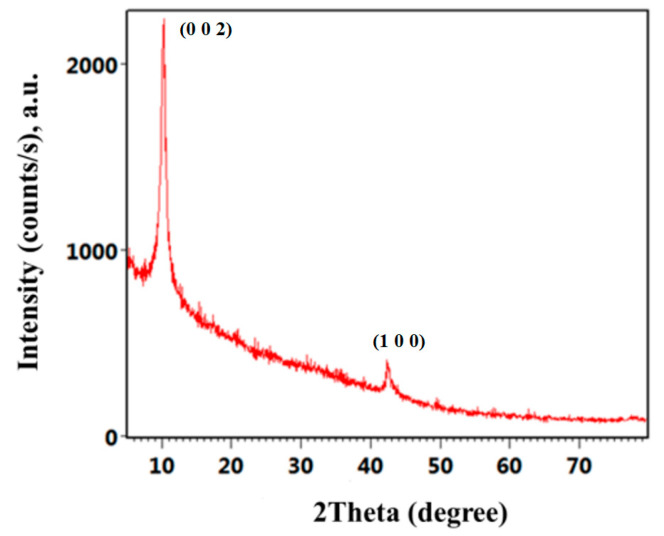
X-ray diffraction (XRD) pattern of graphene oxide nanosheets (nGOs)

Using this version of the Hummers protocol, a high-purity nGO powdery sample was obtained, as the corresponding XRD pattern (Figure 1) evidences the presence of a sharp diffraction maximum at 2θ = 11.3°, corresponding to the (0 0 2) plane of nGOs. According to the literature, the (0 0 2) plane of graphite (corresponding to ~0.34 nm basal spacing) is observed at ~26°, and its shift towards much lower values is due to the successful oxidation [63,64]. In addition, the interlayer spacing observed for the as-synthesized sample (determined by Bragg’s law as ~0.90 nm) confirms the formation of nGOs, as the increased distance between graphitic sheets is due to the successful intercalation of oxygen-containing functional groups (such as hydroxyl, carboxyl, carbonyl, and epoxy groups) and water molecules [65,66]. A second diffraction maximum is noticed at 2θ = 43°, indicating the short-range stacking of graphitic layers [67,68].

**Figure 2:**
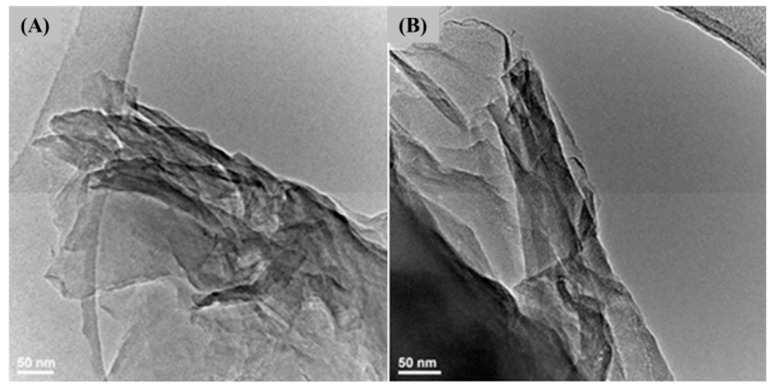
Transmission electron microscopy (TEM) (**A**,**B**) micrographs of graphene oxide nanosheets (nGOs).

Complementarily, TEM images (Figure 2) show the formation of ultra-thin nGOs and confirm the reduced range order in stacked nGO layers (according to previous XRD data), since continuous wrinkled layers with multilamellar and folded structures (seen as darker structures) have been evidenced. These findings are consistent with previous studies that report the formation of highly folded sheets with textured sheet surfaces by the modified Hummers method, as a consequence of highly abundant oxygen-containing functional groups and increased water uptake between graphite layers [62,69,70].

### 2.2. Physicochemical Characterization of PLA-nGO-Zin Coatings

**Figure 3:**
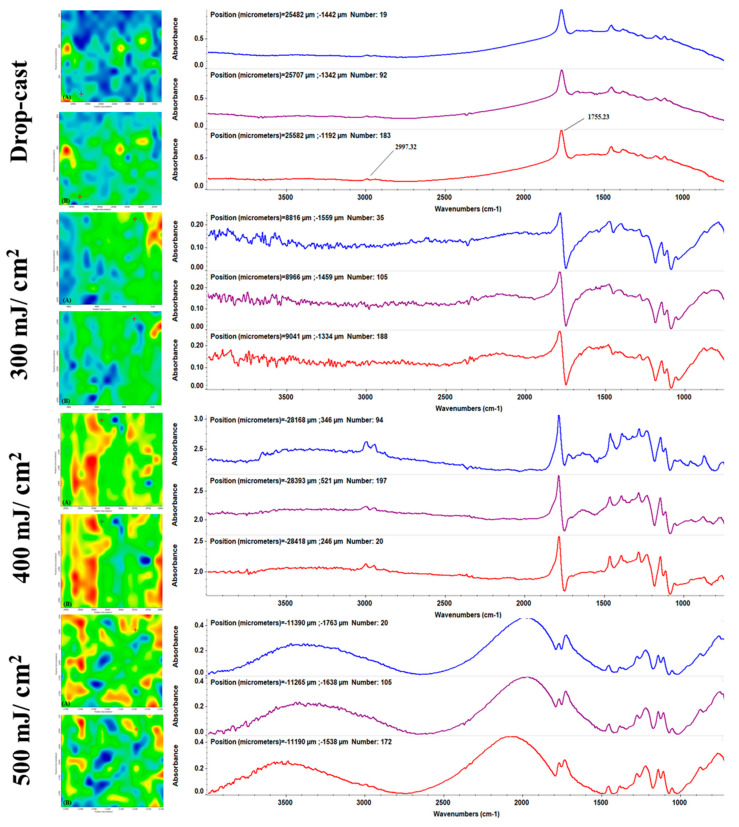
IR maps (**left**) constructed by monitoring the intensity and distribution of C–H (**A**) and C=O (**B**) groups, and corresponding IR spectra (**right**) for PLA-nGO-Zin coatings obtained at different laser fluences.

IRM analysis allows for the concomitant collection of Fourier-transform infrared spectroscopy (FT-IR) spectra (Figure 3, right) and infrared maps (Figure 3, left), thus enabling the facile investigation of coating composition and stoichiometry, and laser-assisted transfer efficiency, respectively.

In this respect, the drop-cast sample (corresponding to the initial mixture of PLA, nGO, and Zin) was used as the reference material. The presence of carbon-containing moieties, originating from both PLA and nGOs, can be noticed at ~1755 cm^−1^ (strong C=O stretching), ~1180 cm^−1^ (C–O–C stretching), and ~1100 cm^−1^ (C–OH stretching) [71,72,73]. Specific PLA functions, assigned to terminal methyl-originating C–H stretching vibrations (~2997 cm^−1^), C–H asymmetric deformation (~1470 cm^−1^), and –CH_3_ bending (~1380 cm^−1^), were also identified [74,75].

The development of biocompatible coatings with application-related physicochemical properties and tuned biofunctionality can be successfully achieved using the MAPLE technique. Some advantages of this versatile laser processing method include the unaltered transfer of small-molecule or macromolecule organics, stoichiometric transfer of inorganic or organic materials, and strong adhesion of the MAPLE coating on the IMD’s surface. Herein, different laser fluences were employed during the MAPLE experiments (300, 400, and 500 mJ/cm^2^), and IRM analysis was used to identify the optimal laser fluence for the MAPLE processing of PLA-nGO-Zin materials. Besides IR spectra (Figure 3, right—corresponding to different points on each sample), infrared maps (Figure 3, left) were also collected by monitoring the absorbance intensity of C–H (A maps) and C=O (B maps) bonds.

Compared to the drop-cast sample, all previously identified IR vibrations were also noticed in the case of MAPLE materials processed at 400 mJ/cm^2^, indicating the compositional integrity and stoichiometry of the coating. A reduced transfer of the composite material is observed for coatings obtained at low laser fluence (300 mJ/cm^2^), with preserved composition, but vibrational signatures of much lower intensity. Conversely, important alterations of the functional groups are observed for composites processed at 500 mJ/cm^2^ laser fluence. It is worth mentioning that most IR absorption maxima of Zin might have been overlapped by the highly abundant carbon-containing constituents (both the polyester and GO), according to the IR spectra of the drop-cast sample. However, in the case of PLA-nGO-Zin coatings obtained at 400 mJ/cm^2^, the additional presence of amide and phosphate moieties within Zin (~1680 and ~1060 cm^−1^, respectively) [76,77,78] may confirm the laser transfer of this cephalosporin antibiotic.

As the color variations in the mapping micrographs (Figure 3, left), ranging from red to blue, are directly related to the high-to-low intensity of monitored absorbance bands, these results confirm that an efficient laser transfer and material distribution of PLA-nGO-Zin composite on the substrate were obtained by using the middle laser fluence.

We have previously reported the successful use of the 300–400 mJ/cm^2^ laser fluence range in obtaining PLA-based coatings for the surface improvement of IMD-related materials [39,49]. Also, similar outcomes regarding the MAPLE processing of GO-based coatings are available, indicating that their stoichiometric transfer can be achieved for reduced-to-moderate laser fluences when using excimer [79,80] or Nd:YAG [81,82,83] laser beams. Given the available literature and the previously discussed IRM data, the middle laser fluence was selected as optimal for the MAPLE processing of PLA-nGO-Zin materials, and thorough investigations were conducted in our study only on the coatings obtained at 400 mJ/cm^2^ laser fluence.

**Figure 4:**
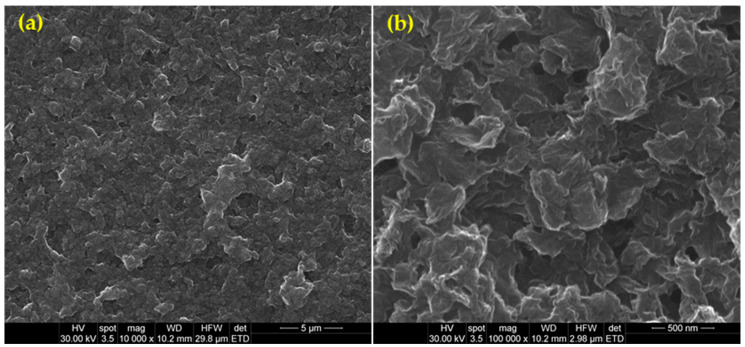
Top-view (**a**,**b**) scanning electron microscopy (SEM) images of PLA-nGO-Zin coatings obtained at 400 mJ/cm^2^ laser fluence.

Consistent with previous studies [79,84], the herein-developed continuous and compact coatings completely cover the substrate. The highly irregular surface appearance of PLA-nGO-Zin coatings (Figure 4a,b) may result from the laser-mediated rearrangement of nGOs, which preserves their irregular and wrinkled aspect and their dimensional range (in compliance with TEM observations). Moreover, the nGOs seem to be individually covered by a thin organic PLA layer with a wavy aspect, while smoother areas can be noticed between larger nGO aggregates. These observations, together with previous IR results, confirm the successful formation of composite and nanostructured PLA-nGO-Zin coatings by performing the MAPLE processing at 400 mJ/cm^2^ laser fluence.

### 2.3. Blood Interaction with PLA-nGO-Zin Coatings

#### 2.3.1. Evaluation of the Hemolytic Potential of PLA-nGO-Zin Coatings

For blood-contacting materials, hemocompatibility is a critical aspect that needs to be considered to select and validate materials that do not exhibit harmful effects on the blood. Hemocompatible materials are selected based on specific assays that highlight the lack of potential of analyzed samples to activate or destroy blood components following material-blood interactions. For this purpose, different aspects can be evaluated, such as the destruction of red blood cells, activation of coagulation via the intrinsic pathway, or pro-inflammatory effects driven by leukocyte activation [85,86]. Therefore, to assess the hemocompatibility of PLA-nGO-Zin-coated samples, their hemolytic and pro-inflammatory potential was investigated following blood interaction.

**Figure 5:**
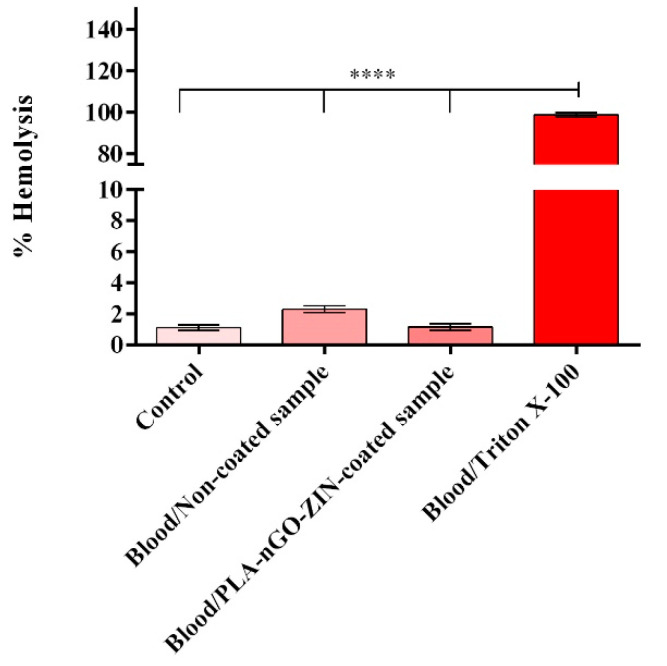
Graphical representation of the hemolysis assay results highlighting the impact of uncoated and PLA-nGO-Zin-coated samples on erythrocyte integrity after 1 h of material–blood sample interaction (**** *p* ≤ 0.0001). The represented data are the mean values of three independent experiments ± S.D.

The ability of PLA-nGO-Zin-coated samples to induce red blood cell lysis was investigated 1 h after interaction with blood samples by the spectrophotometric determination of hemoglobin (released as an indicator of erythrocyte destruction). The obtained results (Figure 5) show that PLA-nGO-Zin-coated samples do not induce damage to the red blood cells’ membranes, as no significant changes are identified between blood samples exposed to the tested materials in comparison with the unexposed control. It is noteworthy that for both the uncoated substrate and PLA-nGO-Zin samples, the hemolysis rate is below the 5% threshold, which defines materials as hemolytic according to the ASTM F756-00 (2000) guidelines [87]. Moreover, although materials presenting a hemolytic index below 5% are considered hemocompatible, these are further characterized as slightly hemolytic and non-hemolytic, with a hemolytic ratio between 2 and 5% or below 2%, respectively. Therefore, as the hemolytic index of non-coated samples is 2.29%, while the hemolytic index of PLA-nGO-Zin-coated samples is 1.15%, these results clearly highlight the great promise of the proposed coating strategy in increasing the material’s blood compatibility.

#### 2.3.2. Assessment of the Pro-inflammatory Potential of PLA-nGO-Zin Coatings by Profiling Cytokine Expression

**Figure 6:**
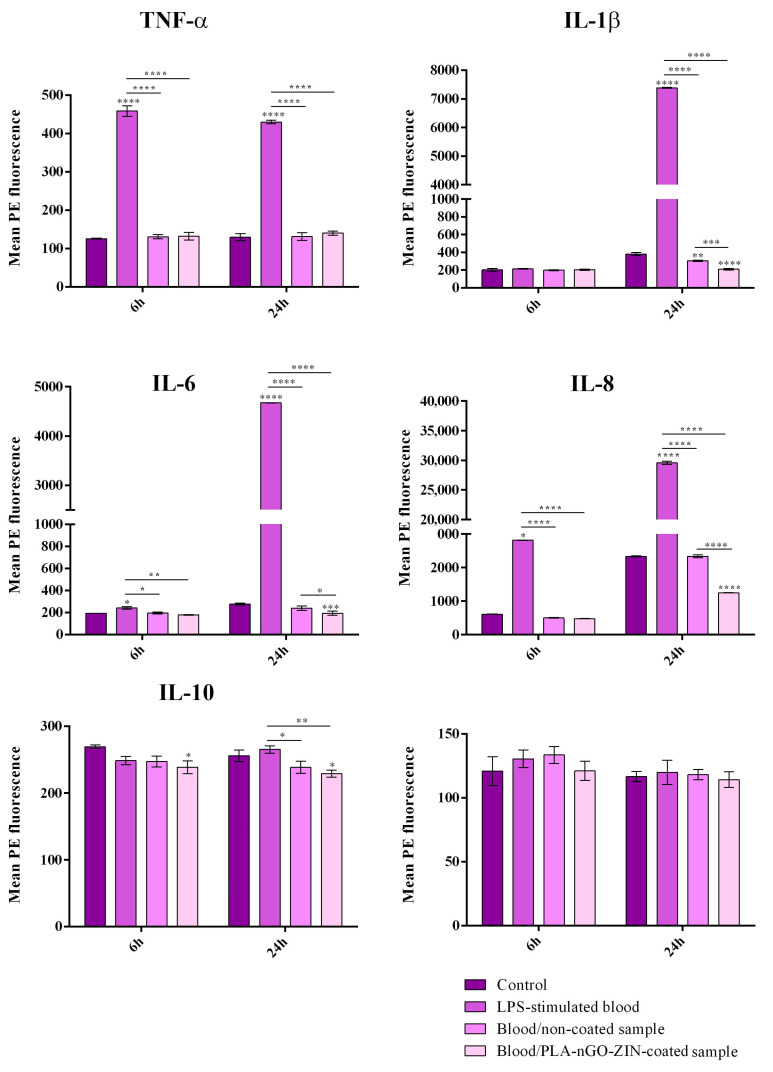
Graphic representation of the IL-8, IL-1β, IL-6, IL-10, TNF-α, and IL-12p70 cytokine levels quantified in recovered supernatants from blood samples which interacted with uncoated and PLA-nGO-Zin samples for 6 h and 24 h. The experimental control was represented by non-stimulated blood samples, while the positive control was represented by LPS-stimulated blood samples. The represented data are the mean values of three independent experiments ± S.D. (* *p* ≤ 0.05; ** *p* ≤ 0.01; *** *p* ≤ 0.001; **** *p* ≤ 0.0001).

To explore the pro-inflammatory potential of PLA-nGO-Zin coatings, the protein expression of six cytokines with an influential role in regulating inflammatory responses was investigated by flow cytometry (Figure 6). The expression of analyzed cytokines is statistically significantly increased in LPS-stimulated blood samples compared to control blood samples, except for IL12p70, where no changes are identified at any of the considered time points. Increasing cytokine production is identified starting with short-term exposure for TNF-α, IL-8, and IL-6, while for the rest of the cytokines, a longer LPS stimulation period is required to trigger significant alterations of the cytokine levels. This enhancement in cytokine production was expected, as LPS is a potent stimulus that activates innate immune cells and triggers in response the gradual production of pro-inflammatory cytokines [88,89].

Regarding our samples, the obtained results show that blood stimulation with PLA-nGO-Zin coatings does not enhance the analyzed cytokine production when compared with the control blood sample. After 24 h of blood–PLA-nGO-Zin sample interaction, a statistically significant decrease in the expression of IL-1β, IL-6, and IL-8 pro-inflammatory cytokines is obtained as compared with cytokine levels quantified in non-stimulated blood samples, results that suggest the impact of PLA-nGO-Zin-coated samples on diminishing the basal levels of these cytokines. Moreover, the expression of these cytokines is statistically significantly lower in blood samples exposed to coated materials than those exposed to uncoated references, showing that deposition of the coating on the reference surface could endorse the original material with potential anti-inflammatory properties. This feature is most likely due to the presence of GO, which has been characterized as a nanomaterial with anti-inflammatory effects, a feature that empowers the use of nGOs in biomedical applications, besides its excellent biocompatibility [90,91].

Consistent with previous studies on nGOs, our results demonstrate the excellent hemocompatibility and moderate anti-inflammatory activity of PLA-nGO-Zin coatings. Owing to their distinctive chemistry and structure, and attractive electrical, mechanical, and thermal peculiarities, nGOs possess a tunable stimulus-responsive ability, which can be explored for fabricating sensitive platforms for the specific and selective detection and imagining of biomarkers and cells in blood samples [92,93,94]. Highly hemocompatible and pro-coagulant nGO-based aerogels crosslinked with gelatin and chitosan (CS), respectively, have been proposed as effective hemostats, their hemostatic performance being superior to commercial products [95]. While nGOs with small lateral dimensions (50 nm to 3 µm) [90] and negatively charged GO quantum dots (<10 nm) [96] selectively inhibit the expression of interleukin genes in macrophages, gold-decorated GO nanocomposites inhibit platelet activation and fibrotic formation, and exhibit important antioxidant and anti-inflammatory effects in macrophages, but also modulate the differentiation of stem cells, thus possessing impressive potential for tissue repair and regeneration [91].

With the aim of developing biofunctional coating materials that modulate the therapeutic outcome of conventional IMDs, impressive results have been reported for GO-embedded polymer materials. For instance, nGOs have been proposed as a mechanical strengthener and anti-corrosion filler for polysaccharide [97,98] and protein [45,99] coatings. Without altering the intrinsic bioactivity of such biopolymers, while promoting and supporting normal events in osteoblast-like cells, these coatings represent suitable candidates for improving the implant-to-host interface of metallic materials. nGOs also act as a mechanical reinforcer and antimicrobial additive for polyesters, facilitating the fabrication of electroactive layered formulations that modulate physiological events in stem and progenitor cells, thus showing great promise for hard [100,101] and soft [102,103] tissue engineering applications. nGO-loaded conductive polymer constructs have been particularly validated as advanced interfaces (or so-called “smart materials”) for guided tissue repair and regeneration [104,105,106].

Reconstruction surgery of knee ligaments with tendon autografts generally requires the use of fixation devices (such as plates, wires, screws, nails, pins, bands, and flexible/adaptable fixation systems), for which the need to improve their post-operative biomechanical performance and overcome their intrinsic bioinertness and microbial susceptibility is thoroughly investigated. For instance, bioresorbable magnesium-based fixators (screws, rods, and wires) have been developed to improve the intra-tunnel bone-to-tendon interface [107,108]. Following their in vivo degradation, such devices promote and stimulate osteogenic events (osteogenic differentiation, mineralization, early new bone formation, late fibrocartilage-like tissue formation, and accelerated intra-tunnel ossification), while alloying and reinforcing elements determine boosted performances in terms of mechanical properties, corrosion behavior, local stability, and controllable degradability. Though comparable tunnel widening has been reported when using bioactive glass or PLA/hydroxyapatite interference screws, the use of bioglass fixation devices results in less aggressive foreign body reaction, superior translational stability, and higher osteointegration and resorption rates [109]. Improving the biomechanics and healing rate of tendon-reconstructed knee ligaments by modulating intra-tunnel ossification has also been reported by using PLA-based tubular interface implants [110,111] and polytetrafluoroethylene-sheathed core bones [112].

Impressive outcomes have been reported when tuning the surface of ligament allografts with nanostructured coatings. By up-regulating the osteogenic and angiogenic differentiation of stem/stromal cells, polyethylene terephthalate (PET) artificial grafts modified with pulsed laser-deposited copper-containing bioglass nanocoatings [113] or plasma-sprayed nano-hydroxyapatite coatings [114] have been validated as biofunctional implants for knee ligament surgery. Besides exhibiting improved hydrophilicity and enhanced biomechanics, the nanocoated ligament implants stimulate new bone formation and neo-vascularization, resulting in faster healing rates. Owing to the multiple roles of silicon and strontium in modulating complex events during bone homeostasis (by stimulating pro-osteoblast action and mineralization, inducing osteogenic differentiation and angiogenesis, and suppressing osteoclastogenesis) [115,116], strontium-enriched silicate nanocoatings have been fabricated for inducing osteogenic activity in PET artificial grafts [117].

### 2.4. Anti-Biofilm Efficiency of PLA-nGO-Zin Coatings

**Figure 7:**
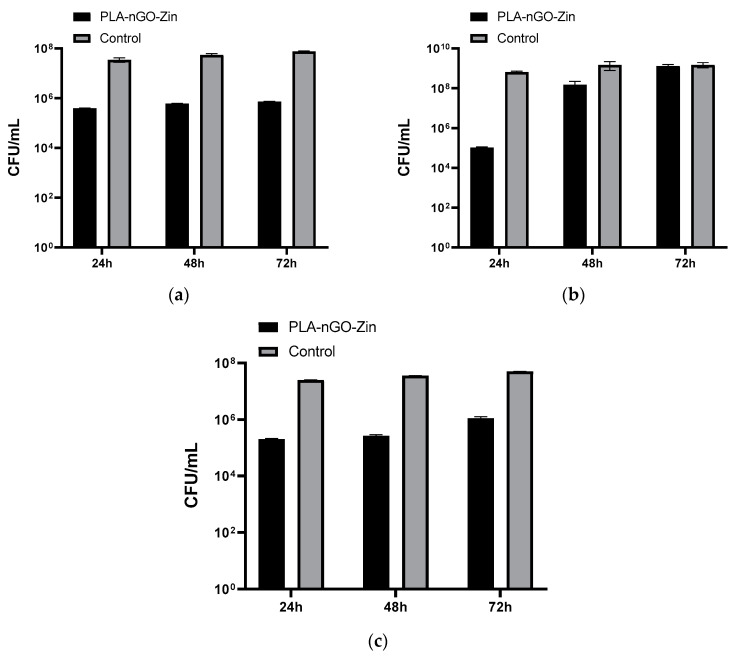
Microbial biofilm development of *E. coli* (**a**), *Ps. aeruginosa* (**b**), and *S. aureus* (**c**) after different incubation periods with PLA-nGO-Zin coatings, expressed as CFU/mL values.

Herein, the ability of PLA-nGO-Zin coatings to alter the development of bacterial biofilms has been evaluated at different time intervals (24, 48, and 72 h) against Gram-negative (*E. coli*, *Ps. aeruginosa*) and Gram-positive (*S. aureus*) pathogens, the results being included in Figure 7.

PLA-nGO-Zin-coated samples determine an important decrease in the bacterial populations of *S. aureus* (Figure 7c) and *E. coli* (Figure 7a) compared to control specimens, with a sustained diminution of the CFU/mL values of ~2 orders of magnitude (logs). These results confirm the preserved antimicrobial efficiency of the intravenous ceftaroline fosamil (a fifth-generation cephalosporin prodrug with broad-spectrum activity) [35,118] following MAPLE processing. Further, a comparable and sustained inhibition of biofilms is noticed for both bacteria, indicating the prolonged efficiency of PLA-nGO-Zin nanostructured coatings to alter the development of bacterial biofilms at different stages.

Still, the most important effect in inhibiting biofilm development is observed for the *Ps. aeruginosa* strain (Figure 7b), in the case of short-time contact, with an inhibitory efficiency of close to 4 logs. After 48 h, the bacterial population is reduced by only one order of magnitude, and PLA-nGO-Zin coatings eventually lose their anti-biofilm ability after 72 h. These results demonstrate the enhanced ability of PLA-nGO-Zin coatings to interfere with *Ps. aeruginosa* biofilm during the contamination, colonization, and early maturation phases.

Our findings are compliant with other studies that report the early-stage efficiency of Zinforo™ against bacterial biofilms [41,119]. More than that, we report the prolonged anti-biofilm efficiency of PLA-nGO-Zin coatings against *S. aureus* and *E. coli*, which may be related to the synergistic antimicrobial effects of Zinforo™ and nGO. Like other nanomaterials, GO exhibits important size-related antimicrobial effects by mechanically disrupting the integrity of bacterial cell membranes [45]. Moreover, due to the functional groups present on its surface, GO can electrostatically interact with the phospholipids forming the bacterial cell membrane [120,121]. Also, GO-mediated physical demolition and chemical oxidation lead to the generation of reactive species, which cause microbial death and decreased microbial resistance [54].

Incorporating graphene derivatives into PLA matrices results in reinforcing the polyester’s mechanical behavior, inducing antibacterial effects by the inhibition of Gram-negative and Gram-positive bacterial proliferation, while lacking cytotoxicity and improving the adhesion and spreading of healthy normal cells, being thus evaluated as a promising solution for biomedical applications [59]. Also, the addition of GO within PLA–polyurethane [122,123] and CS-PLA [124,125] composites has also led to the successful fabrication of bioactive nanostructured constructs with excellent biocompatibility, strong antimicrobial effects, and good thermomechanical features.

Our results align with other studies regarding the development of unconventional strategies to modulate the microbial susceptibility of fixation devices used in knee ligament reconstruction. Resorbable hyaluronan/PLA hydrogel loaded with vancomycin has been reported as an effective and safe coating for tendon autografts, acting as a prophylactic solution for periprosthetic infections [126]. Also, electroactive nanofibrous scaffolds of PLA/graphite nanoplatelets modified with silver nanoparticles, exhibiting good thermomechanical behavior and reduced degradation, have been developed as a functional ligament substitute that exerts local antibacterial effects [127].

## 3. Materials and Methods

### 3.1. Materials

Sigma-Aldrich (Merck Group, Darmstadt, Germany) was the supplier of all reagents used during the synthesis of nanosheets and nanostructured coatings, namely H_2_SO_4_, K_2_S_2_O_8,_ P_2_O_5_, KMnO_4_, H_2_O_2_, HCl, dimethyl sulfoxide (DMSO), and polylactic acid (PLA). Analytical-grade pure chemicals were used throughout the experiments.

The same supplier (Sigma/Merck) provided most reagents used for the in vitro evaluation of obtained coatings (otherwise, the provider was accordingly specified during the protocol). Bacterial strains were obtained from the American Type Culture Collection (ATCC, Manassas, VA, USA).

### 3.2. Synthesis Methods

#### 3.2.1. Synthesis of Graphene Oxide (GO) Nanomaterial

Pre-oxidized graphite was firstly obtained by dispersing the graphite powder in concentrated sulfuric acid mixed with potassium persulfate and phosphorous pentoxide under stirring at 80 °C. After reaching room temperature (RT), the mixture was washed several times (until it achieved a neutral pH), then subjected to filtration and dried at 80 °C for 24 h. The as-obtained pre-oxidized graphite powder was re-dispersed in concentrated H_2_SO_4_ solution by stirring in an ice bath, then potassium permanganate was slowly added in the beaker at a temperature close to 0 °C. Further, the mixture was magnetically stirred at 35 °C for 2 h and stirred at 80 °C for 2 h, washed by stirring for 15 min, and finally stirred for 2 h with 30% hydrogen peroxide solution. The as-resulted precipitate was subjected to vacuum filtration, washed with 3% hydrochloric acid solution until it reached a neutral pH, then air-dried at 60 °C for 24 h. A modified Hummers method [128,129] was used to synthesize the graphene oxide nanosheets (nGOs).

#### 3.2.2. Synthesis of PLA-nGO-Zin Coatings

For the deposition of coatings based on polylactic acid (PLA), graphene oxide nanosheets (nGOs), and ceftaroline fosamil cephalosporin (Zinforo™, Zin), substrates with 1 cm^2^ area of double-side-polished (1 0 0) silicon (for physicochemical investigation), glass, and titanium (for in vitro evaluation) were used. Before surface modification by means of matrix-assisted pulsed laser evaporation (MAPLE) [49,130], all substrates were successively cleaned with acetone, ethanol, and deionized water in an ultrasonic bath (15 min each step), then dried under a high-purity nitrogen jet.

Suspensions with 5% concentration of PLA, nGOs, and Zin (10:5:1 wt.%) prepared in DMSO were transferred in copper holders and immersed in liquid nitrogen for 30 min. The as-obtained solid targets were irradiated with a COMPexPro 205 Lambda Physics source (KrF* excimer laser beam with λ = 248 nm, τ_FWHM_ = 25 ns, and 10 Hz repetition frequency), purchased from Coherent (Göttingen, Germany). During MAPLE experiments, the following parameters were constant: RT and 0.1 Pa pressure inside the deposition chamber, 5 cm target-to-substrate distance, and 0.4 Hz target rotation. For each experiment, 50,000 laser pulses were applied at different laser fluences (300, 400, and 500 mJ/cm^2^).

### 3.3. Physicochemical Investigation Methods

#### 3.3.1. Characterization of GO Nanosheets (nGOs)

The purity, crystalline nature, and microstructure of the as-synthesized powdery sample were investigated by X-ray diffraction (XRD) and transmission electron microscopy (TEM).

For XRD analysis, an Empyrean diffractometer with Cu_Kα_ radiation from PANalytical (Almelo, The Netherlands), equipped with a hybrid monochromator for Cu and a PIXcel3D detector, was used. Scans were collected on the mildly ground powder, using Bragg–Brentano geometry between 5–80° diffraction angles, with 0.5° incidence angle, under step scan mode (0.04° scanning step size and 3 s acquisition time per scanning step).

TEM investigation was performed using the Tecnai^TM^ G2 F30 S-TWIN high-resolution equipment from Thermo Fischer Scientific (former FEI, Hillsboro, OR, USA). Data collection was performed in transmission mode, with point and line resolutions of 2 Å and 1 Å, respectively. Before analysis, small amounts of the as-synthesized powdery sample were dispersed in ethanol under sonication for 15 min, then placed onto the carbon-coated copper grid and dried at RT.

#### 3.3.2. Characterization of PLA-nGO-Zin Coatings

Relevant compositional, structural, and morphological aspects of the MAPLE-processed samples were provided by complementary infrared microscopy (IRM) and scanning electron microscopy (SEM) investigations.

A Nicolet iN10 MX FT-IR microscope from Thermo Fischer Scientific (Waltham, MA, USA) was used for IRM analysis to collect IR spectra and IR maps. The scans (32 measurements per sample) were recorded in the transmission mode in the 4000–750 cm^−1^ wavenumber range (with 4 cm^−1^ resolution), and then processed with the OmincPicta 8.0 software (Thermo Fischer Scientific).

For SEM investigation, Inspect S equipment from FEI Company (Thermo Fischer Scientific, Hillsboro, OR, USA) was used. Micrographs were collected using the secondary electron beam with 30 keV after capping all samples with a thin conductive layer.

### 3.4. Biological Evaluation of PLA-nGO-Zin Coatings

Peripheral blood samples were used to investigate the blood response to PLA-nGO-Zin-coated samples. The blood samples were collected by qualified medical personnel using EDTA blood collection vacutainers (Becton Dickinson, Franklin Lakes, NJ, USA) from healthy donors after obtaining their written informed consent. Fresh blood samples were used for all experiments within the first hour after withdrawal.

To reveal if PLA-nGO-Zin-coated samples triggered hemolysis, blood samples were centrifuged to recover erythrocytes, which were further washed in 150 mM NaCl (Sigma/Merck). The obtained pellet was re-suspended in PBS (Sigma/Merck) to reach a final volume of 5 mL, and the obtained solution was further diluted to 1:50 with PBS. Pristine (uncoated) glass slides and PLA-nGO-Zin-coated samples were transferred to 24-well plates, incubated for 1 h at 37 °C on a plate shaker, and centrifuged. Then, 20% Triton X-100 (Sigma/Merck) was added to the positive control wells, while the negative control wells were represented by simple erythrocyte solutions. After 1 h, samples were centrifuged, and the resulting supernatants were transferred to 96-well plates and measured at 492 nm to determine their optical densities (ODs) using the FlexStation III multimodal reader from Molecular Devices (San Jose, CA, USA). To express hemolysis as a percentage, the following formula was used [131,132], with the OD of the negative control being the sample with minimal lysis, and the OD of the positive control being the sample where the maximal lysis was registered:

$$hemolysis\left(\%\right)=\frac{OD\;sample-OD\;negative\;control}{OD\;positive\;control-OD\;negative\;control}\times 100$$

The pro-inflammatory potential of PLA-nGO-Zin-coated samples was explored by measuring at different post-contact time points (6 h and 24 h) the levels of the following pro-inflammatory cytokines in blood samples: Interleukin-8 (IL-8), Interleukin-1β (IL-1β), Interleukin-6 (IL-6), Interleukin-10 (IL-10), Tumor Necrosis Factor (TNF-α), and Interleukin-12p70 (IL-12p70). In this view, the tested materials (both uncoated and PLA-nGO-Zin-coated samples) were immersed for 6 h and 24 h in fresh blood samples on a shaker at 37 °C. At the pre-set experimental time points, tested materials were retrieved from the blood samples, which were then centrifuged to recover blood serum that was subsequently employed for cytokine quantification. Clear blood samples were used as negative controls, while blood samples stimulated with 10 μg/mL of lipopolysaccharide (LPS) from *Escherichia coli* O111:B4 (Sigma/Merck) were employed as positive controls. To quantify the cytokine levels in all experimental samples, the BD™ Cytometric Bead Array (CBA) Human Inflammatory Cytokines Kit (Becton Dickinson) was used [131,133]. Briefly, the capture bead mix was prepared as described by the manufacturer, and 50 µL of this solution was mixed with 50 µL of the tested samples, and then incubated for 1.5 h at RT in the dark. After a wash step, samples were further mixed with 50 µL of the Human Inflammatory Cytokines PE Detection Reagent and additionally incubated for 1.5 h at RT in the dark. The bead pellets were recovered by centrifugation, re-suspended in 300 µL of wash buffer provided in the kit, and immediately analyzed using a Cytoflex flow cytometer (Beckman Coulter, Brea, CA, USA). Data were acquired and analyzed by CytExpert v2.3, while GraphPad Prism software v6.07 (GraphPad, San Diego, CA, USA) was employed to represent the obtained results graphically and statistically (as the mean value of triplicate experiments ± standard deviation, S.D., with two-way ANOVA algorithm).

### 3.5. Microbiological Evaluation of PLA-nGO-Zin Coatings

To evaluate the efficiency of MAPLE-processed coatings against monospecific biofilms, the development of Gram-positive (*Staphylococcus aureus* ATCC^®^ 25923) and Gram-negative (*Escherichia coli* ATCC^®^ 15224, *Pseudomonas aeruginosa* ATCC^®^ 27853) bacterial biofilms in the presence of PLA-nGO-Zin-coated samples was assessed for different time points (24 h, 48 h, and 72 h).

UV-sterilized uncoated (pristine) and PLA-nGO-Zin-coated substrates were transferred to sterile 24-well plates containing 1 mL of Luria–Bertani (LB) broth (Thermo Fischer Scientific), then inoculated with 10 μL of 0.5 McFarland standard density microbial suspensions. The prepared plates were incubated at 37 °C for 24 h, then the culture media was removed, and the samples were washed with sterile phosphate-buffered saline (PBS, Sigma/Merck). The as-treated samples were further placed in new sterile plates containing fresh nutritive broth and incubated at 37 °C for 24 h, 48 h, and 72 h. After incubation, samples were gently washed with PBS and transferred in 1.5 mL centrifuge tubes containing sterile PBS. All specimens were successively vortexed (30 s) and sonicated (10 s) to detach the biofilms and obtain the biofilm-embedded microbial cell suspensions. Serial ten-fold dilutions were further performed, and the final microbial suspensions were seeded on LB agar plates (Thermo Fischer Scientific) to evaluate the colony-forming units (CFU/mL) by viable cell count assay [49,134]. Antimicrobial results were processed with the GraphPad Prism software (GraphPad v6.07), using the one-way ANOVA statistical tool.

## 4. Conclusions

Fabricating biocompatible nanosized or nanostructured coatings is an attractive and flexible strategy to modulate the microbial susceptibility of implantable medical devices. More than providing protection to metallic surfaces, the thermomechanical and barrier properties, bioactivity, and pathogenic susceptibility of polylactide (PLA)-based coatings can be tuned by reinforcement with graphene oxide (GO) nanomaterials. Also, the versatile biochemistry and thermomechanics of PLA films provide indisputable advantages for fabricating active coatings that induce or potentiate local antimicrobial effects.

Herein, biodegradable coatings based on PLA, GO nanosheets (nGOs), and Zinforo™ (Zin-a broad-spectrum cephalosporin prodrug) were developed by laser processing. Following a comparative IRM analysis, the 400 mJ/cm^2^ laser fluence was selected for the transfer of PLA-nGO-Zin coatings. Continuous and compact thin coatings with irregular surfaces were thus obtained, the nGOs preserving the initial wrinkled aspect and folded structure while being uniformly embedded within the PLA matrix.

Following interactions with fresh blood samples, biological results have evidenced the excellent hemocompatibility of PLA-nGO-Zin coatings, the hemolytic index being 1.15% (corresponding to a non-hemolytic material). The anti-inflammatory activity of as-proposed original coatings has also been reported after a 24 h contact with blood samples, as the PLA-nGO-Zin-coated specimens significantly lowered the basal levels of IL-1β, IL-6, and IL-8 pro-inflammatory cytokines.

The microbiological data demonstrate the sustained and extended (up to 3 days) anti-biofilm efficiency of PLA-nGO-Zin coatings against *S. aureus* and *E. coli* biofilms, while a more drastic inhibitory action was shown during the incipient development of *Ps. aeruginosa* biofilm.

The highly hemocompatible PLA-nGO-Zin nanostructured coatings, exhibiting anti-inflammatory activity and important anti-biofilm efficiency, are suitable candidates for the surface modification of implantable metallic devices, representing an attractive strategy for the local prevention and limitation of biofilm-associated periprosthetic infections.

## Data Availability

Data are available from the authors on request.
